# The Expression of Formyl Peptide Receptor 1 is Correlated with Tumor Invasion of Human Colorectal Cancer

**DOI:** 10.1038/s41598-017-06368-9

**Published:** 2017-07-19

**Authors:** Shu-Qin Li, Ning Su, Ping Gong, Hai-Bo Zhang, Jin Liu, Ding Wang, Yan-Ping Sun, Yan Zhang, Feng Qian, Bo Zhao, Yang Yu, Richard D. Ye

**Affiliations:** 1 School of Pharmacy, Shanghai Jiao Tong University, 800 Dongchuan Road, Shanghai, 200240 China; 2 School of Pharmacy, Ruijin Hospital Affiliated to Shanghai Jiao Tong University School of Medicine, 197 Ruijin Road (No.2), Shanghai, 200025 China; 3 Department of General Surgery, Shanghai Chang Zheng Hospital, Second Military Medical University, 415 Fengyang Road, Shanghai, 200003 China; 4 Institute of Chinese Medical Sciences, University of Macau, Macau, 999078 China

## Abstract

Formyl peptide receptors (FPRs) are G protein-coupled chemoattractant receptors expressed mainly in phagocytic leukocytes. High expression of FPRs has also been detected in several cancers but the functions of FPR1 in tumor invasion and metastasis is poorly understood. In this study, we investigated the expression of FPRs in primary human colorectal cancer (CRC) and analyzed the association of FPRs expression with clinicopathological parameters. The levels of FPRs mRNA, especially those of FPR1, were significantly higher in colorectal tumors than in distant normal tissues and adjacent non-tumor tissues. FPR1 mRNA expression was also associated with tumor serosal infiltration. FPR1 protein expression was both in the colorectal epitheliums and tumor infiltrating neutrophils/macrophages. Furthermore, the functions of FPR1 in tumor invasion and tissue repair were investigated using the CRC cell lines SW480 and HT29. Higher cell surface expression of FPR1 is associated with significantly increased migration in SW480 cells compared with HT29 cells that have less FPR1 membrane expression. Finally, genetic deletion of *fpr1* increased the survival rate of the resulting knockout mice compared with wild type littermates in a mouse model of colitis-associated colorectal cancer. Our data demonstrate that FPR1 may play an important role in tumor cell invasion in CRC patients.

## Introduction

Colorectal cancer (CRC) is one of the most common malignancies in the world^1^. In 2015 alone, CRC accounted for more than 750,000 deaths with 1.48 million new cases^2^. Several risk factors are associated with CRC progression, including aging, genetic aberrations including mutations, and chronic intestinal inflammation^1, 3–5^. As a type of highly invasive carcinomas, CRC has high rates of lymphatic duct invasion, venous invasion, and lymph node metastasis^6^. Metastatic colorectal cancer (mCRC) is a major cause of mortality in CRC patients^7^. However, the underlying mechanisms for CRC tumor invasion and metastasis have not been fully elucidated.

Formyl peptide receptors (FPRs) are a group of seven transmembrane domains, G protein-coupled receptors^8^. In humans, three distinct FPRs (FPR1, FPR2, and FPR3) are encoded by three identified genes, *FPR1*, *FPR2*, and *FPR3* ^9, 10^. FPRs are known to play important roles in host defense and inflammation. In phagocytes, binding of both exogenous and endogenous agonists to FPRs triggers a G protein-mediated signaling cascade, that leads to chemotaxis, calcium flux, phagocytosis, and release of pro-inflammatory mediators^11, 12^. Although FPRs are expressed mainly on phagocytic leukocytes such as neutrophils, monocytes, and macrophages^8^, these receptors have also been detected on cells of non-hematopoietic tissues and cell types including epithelial cells, smooth muscle, endothelial cells and neurons^13^. Recently, FPRs have been found in several types of human cancer tissues and cells, such as highly malignant glioblastoma (GBM) cells^14, 15^, astrocytoma cells^16^, gastric cancer^17, 18^, lung cancer^19^, breast cancer^20^, neuroblastoma cells^21^, and melanoma skin cancer^22^. The potential roles for FPRs in cancer cells have only been investigated in a limited number of disease models and the results suggest that FPRs exert different functions in tumor growth and angiogenesis in different cancer histotypes^23^. However, the level of expression and the role for FPRs in human colorectal cancer remain unclear.

In this study, we investigated the expression of FPRs in primary human colorectal cancer and analyzed the association of FPRs expression with clinicopathological parameters. We found that the mRNA level of FPRs, especially that of FPR1, was significantly higher in colorectal tumors than in distant normal tissues and adjacent non-tumor tissues. Furthermore, FPR1 mRNA expression was associated with the size of tumor and serosal infiltration. Of note, FPR2 expression correlated with the location of the tumor, and FPR3 was not significantly associated with any of these characteristics except for the expression of FPR2. These results suggest that the expression of FPR1 may play a more important role in tumor invasion in CRC patients compared with FPR2 and FPR3. The protein expression level and distribution of FPR1 were further investigated in human CRCs, and the biological functions of this receptor were examined in CRC cell lines and FPR1 gene knock-out (*fpr1* ^−/−^) mice.

## Results

### Baseline characteristics of the colorectal cancer patients

Twenty CRC patients were enrolled in this study, with 8 (40%) males and 12 (60%) females. Their age ranged from 39 to 78 years (mean age, 57 years). Twelve patients had colon cancers (60%) and eight patients had rectal cancers (40%). According to the TNM (Tumor Node Metastasis) classification, all patients had T1, T2, T3, or T4 cancers, with 4 patients (20%) classified as having poorly differentiated and 16 (80%) as good or moderately differentiated cancers. One of these patients (5%) had distant metastasis (M1). The demographic and disease characteristics of these patients are summarized in Supplementary Table S1.

### Increased FPR1 expression was correlated with serosal infiltration

**Figure 1:**
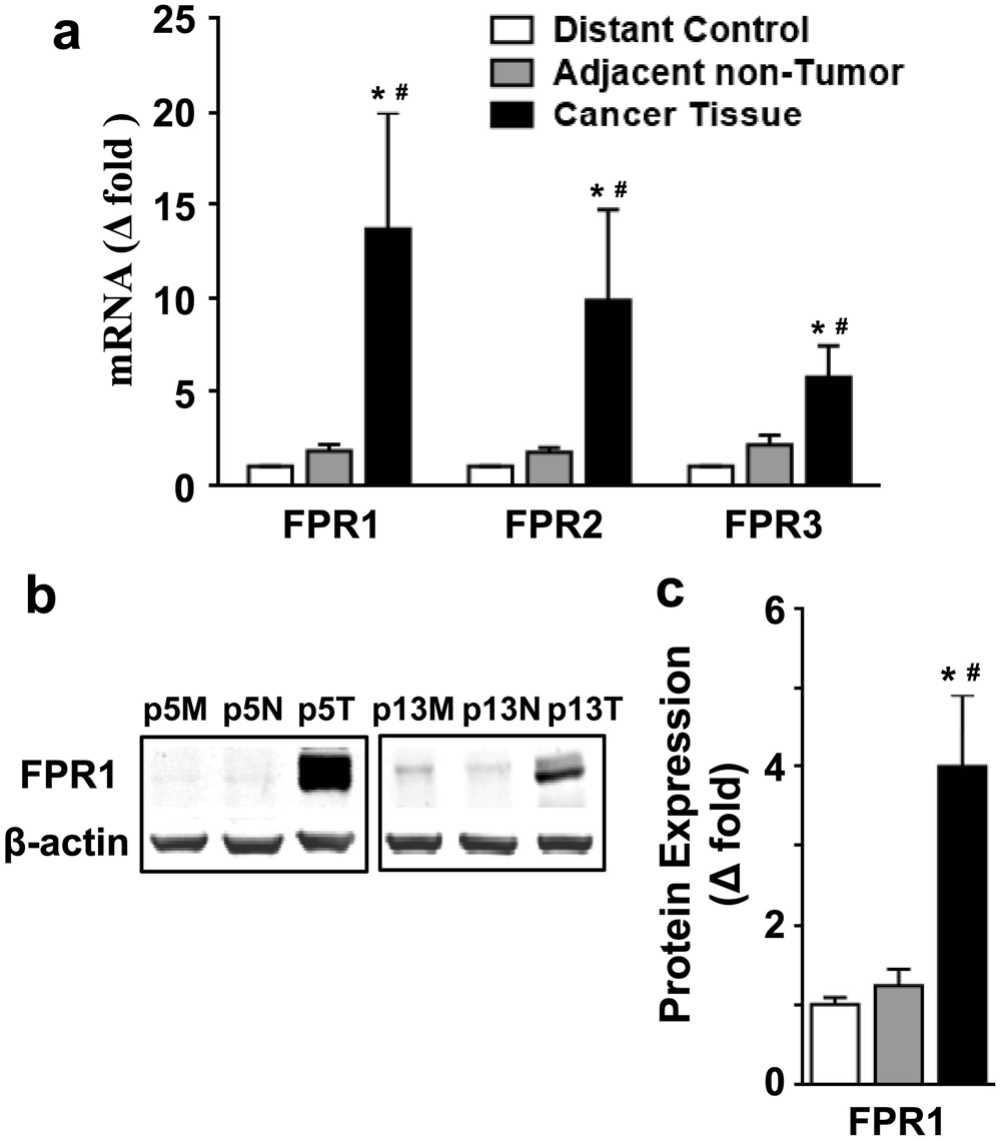
Expression of FPRs was increased in CRC tissues. (**a**) The levels of FPR mRNA in CRC tissues, distant control, and adjacent non-tumor tissues were examined with real-time quantitative PCR. The relative transcript expression was calculated as 2^−ΔΔCT^ and was normalized against GAPDH/PPIA. (**b**) The tissue homogenates were analyzed by western blotting for the expression of FPR1. Two representative sample blots (p5 and p13) are shown. M, distant mormal tissue; N, adjacent non-tumor tissue; T, cancer tissue. The full-length blots were presented in Supplementary Fig. S3. (**c**) The blots were quantified densitometrically and the relative immunoreactivities are shown. All data shown are means ± SEM. *p < 0.05 compared with distant control; ^#^ *p* < 0.05 compared with adjacent non-tumor tissue.

To evaluate FPRs expression in the CRC, real-time qPCR was performed with samples collected from distant normal tissues, adjacent non-tumor tissues and cancer tissues. The GAPDH/PPIA ratio was used as a normalization control^24^. We found that the mRNA levels of FPRs, especially those of FPR1, were significantly higher in colorectal tumors than in distant normal tissues and adjacent non-tumor tissues (*p* < 0.05). There was no significant difference in gene expression of FPRs between distant normal tissues and adjacent non-tumor tissues (Fig. 1a).

Since the mRNA levels of FPRs in CRC tissues were markedly elevated, we next examined the correlation between FPRs expression and clinicopathological characteristics which included sex, age, stage and location of tumor, tumor size, differentiation, lymphatic invasion, serosa infiltration, and distant metastasis. Changes in FPRs mRNA expression were deemed either increased when the ratio of their levels in tumors against distant normal tissues was no less than 2.0, or decreased when the ratio was no more than 0.5, or unaltered when the ratio was between 0.5–2.0 (Table 1). The results showed that FPR1 mRNA expression level was associated with tumor size (*p* < 0.05) and serosal infiltration (*p* < 0.001). The FPR2 expression level was related with the location of the tumor (*p* < 0.05). The expression level of FPR3 was not significantly associated with any of these characteristics, except for the expression of FPR2 (*p* < 0.05) (Table 1). These results suggest that the expression level of FPR1, but not that of FPR2 and FPR3, may be functionally related to tumor cell invasion in CRC patients. We further examined the expression of FPR1 at the protein level using western blot with an anti-FPR1 antibody, normalized against the expression level of β-actin. Significant upregulation of FPR1 protein level was found in CRC tissue compared with distant normal tissues and adjacent non-tumor tissues (Fig. 1b and c), which was consistent with its mRNA expression profile.

**Table 1:** FPRs and clinicopathological characteristics.

Parameters	No. of patients n (%)	Exp. of FPR1 n (%)			*p* value	Exp. of FPR2 n (%)			*p* value	Exp. of FPR3 n (%)			*p* value
		↑	−	↓		↑	−	↓		↑	−	↓	
All patients	20 (100)	13 (65)	3 (15)	4 (20)		11 (55)	8 (40)	1 (5)		11 (55)	7 (35)	2 (10)	
Location of tumor													
Colon	12 (60)	9 (69)	1 (33)	2 (50)	0.47	10 (89)	1 (9)	1 (100)	**0**.002**	8 (87)	3 (43)	1 (50)	0.45
Rectum	8 (40)	4 (31)	2 (67)	2 (50)		1 (11)	7 (91)	0 (0)		3 (13)	4 (57)	1 (50)	0.33
Size of tumor													
≤3	3 (15)	0 (0)	2 (67)	1 (25)	**0**.0272*	0 (0)	3 (38)	0 (0)	0.18	0 (0)	2 (29)	1 (50)	0.43
3.1–6	12 (60)	9 (69)	0 (0)	3 (75)		7 (64)	4 (50)	1 (100)		7 (64)	4 (57)	1 (50)	
>6	5 (25)	4 (31)	1 (33)	0 (0)		4 (36)	1 (12)	0 (0)		4 (36)	1 (14)	0 (0)	
Serosa infiltration													
Yes	17 (85)	13 (100)	3 (100)	1 (58)	**0**.0009***	10 (89)	6 (25)	1 (100)	0.58	10 (89)	5 (71)	2 (100)	0.55
No	3 (15)	0 (0)	0 (0)	3 (42)		1 (11)	2 (75)	0 (0)		1 (11)	2 (29)	0 (0)	
Exp. of FPR2													
↑	11 (55)									8 (87)	3 (43)	0 (0)	**0**.0179*
−	8 (40)									3 (13)	4 (57)	1 (50)	
↓	1 (5)									0 (0)	0 (0)	1 (50)	

Exp., Expression; *P < 0.05; **P < 0.01; ***P < 0.001.

### FPR1 expression in colorectal epitheliums and in tumor infiltrating neutrophils/macrophages

**Figure 2:**
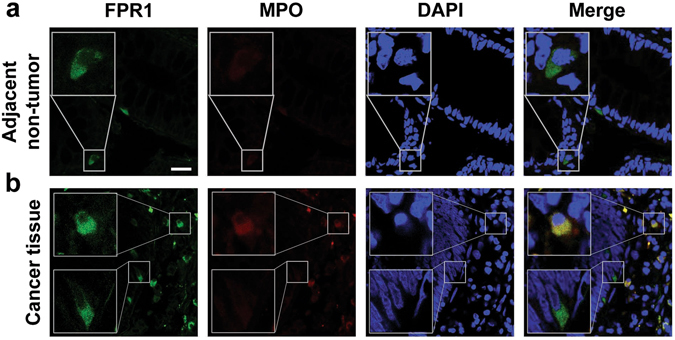
Immunofluorescence staining of FPR1 in human CRC tissues. Frozen sections of the adjacent normal tissues (**a**) and CRC tissues (**b**) were stained by immunofluorescence for the expression of FPR1 (FITC, green) and MPO (Cy3, red), as described in *Materials and Methods*. Cell nuclei were stained with DAPI (blue). Images shown are representative of three independent experiments with similar results. Scale bar, 25 µm.

Since the expression of FPR1 was significantly increased in CRC tissues compared with adjacent non-tumor tissues, we further determined the cellular source of FPR1 in these tissues using immunofluorecence staining. Previous work showed that FPR1 is present on certain epithelial cells (including follicular cells of the thyroid and cortical cells of the adrenal gland) and infiltrating leukocytes such as neutrophils and monocytes/macrophages^13^. Thus, we examined whether FPR1 was expressed in the colorectal epithelium and tumor-infiltrating neutrophils and macrophages. As shown in Fig. 2a, few colorectal epithelial cells in the adjacent normal tissue expressed FPR1. In CRC tissues, both colorectal epithelial cells (MPO negative) and tumor infiltrating myeloid cells (MPO positive) expressed FPR1 (Fig. 2b). These results suggest that the increased expression of FPR1 in CRC tissues may come from both colorectal epithelial cells and tumor-infiltrating myeloid cells. As the expression of FPR1 in the infiltrating leukocytes are well expected and may be an important part of the tumor microenvironment, the increased expression of FPR1 in CRC epithelial cells is of interest to us and suggests a correlation with the invasiveness of CRC.

### FPR1 was expressed in colorectal cancer cell lines

**Figure 3:**
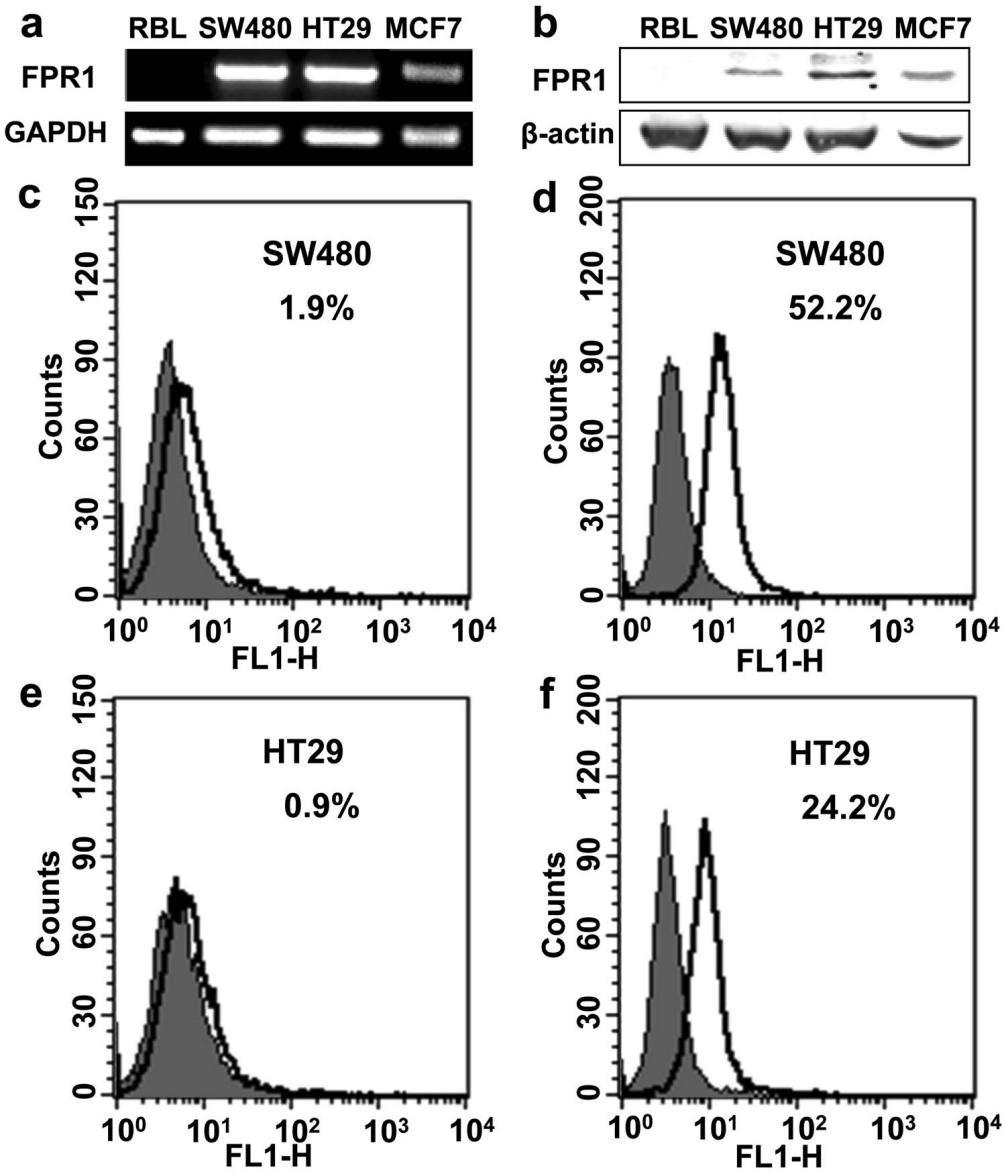
Expression of FPR1 in colorectal cancer cell lines. The mRNA transcripts (**a**) and protein expression (**b**) of FPR1 in SW480 and HT29 were analyzed by agarose gel electrophoresis and western blotting, respectively. The full-length gels and blots were presented in Supplementary Fig. S4a ﻿﻿and﻿ b. (**c**–**f**) Flow cytometry was used to confirm the distribution of FPR1 expression in these two cell lines. The cells were treated with or without membrane-penetration solution to detect the total protein expression (**d**,**f**) and membrane expression (**c**,**e**).

To study the potential role of FPR1 in a CRC model without concerns over individual variations, two human CRC cell lines, SW480 and HT29, were used. We firstly examined the expression of FPR1 in these two cancer cells using qPCR and western blotting. The results showed that FPR1 was moderately expressed in both SW480 and HT29 not only at mRNA level (Fig. 3a) but also at protein level (Fig. 3b). In addition to western blotting, flow cytometry was used to confirm the distribution of FPR1 expression in these two cell lines. The cells were treated with or without membrane-penetration solution to detect total protein expression and membrane expression of FPR1. The result showed a moderate level of total FPR1 expression in both SW480 cells and HT29 cells (Fig. 3d and f, with membrane-penetrating solution). A small amount of FPR1 was expressed on the cell surface of SW480 cells (Fig. 3c), while less expression of FPR1 was recorded on HT29 cell surface (Fig. 3e).

### FPR1 activation promoted SW480 cell migration and invasion

**Figure 4:**
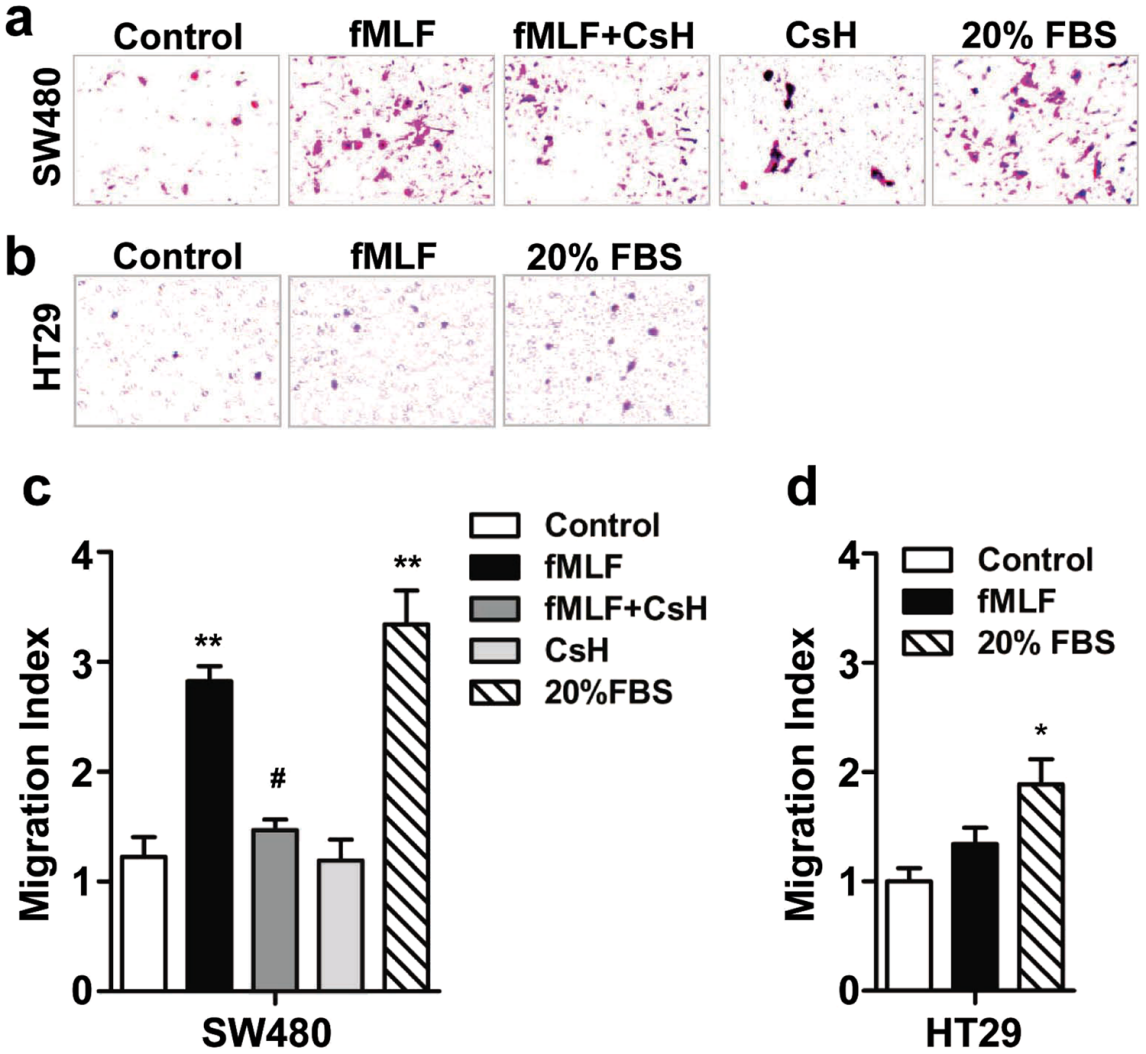
FPR1 activation promoted SW480 cell migration. SW480 (**a**) and HT29 (**b**) were treated with fMLF (1 μM) or 20% FBS (positive control) for 12 h with or without a 15 min pretreatment with cyclosporine H (1 μM). The cell migration was examined using a 48-well Boyden chamber. Representative images of migrated cells (SW480 and TH29) on membrane filters were shown in (**a** and **b**) and quantified data were shown in (**c** and **d**), respectively. *p < 0.05 compared with control, ^#^ *p* < 0.05 compared with the cells treated with fMLF. All the data shown are mean ± SEM from three separate experiments, each in triplicates.

**Figure 5:**
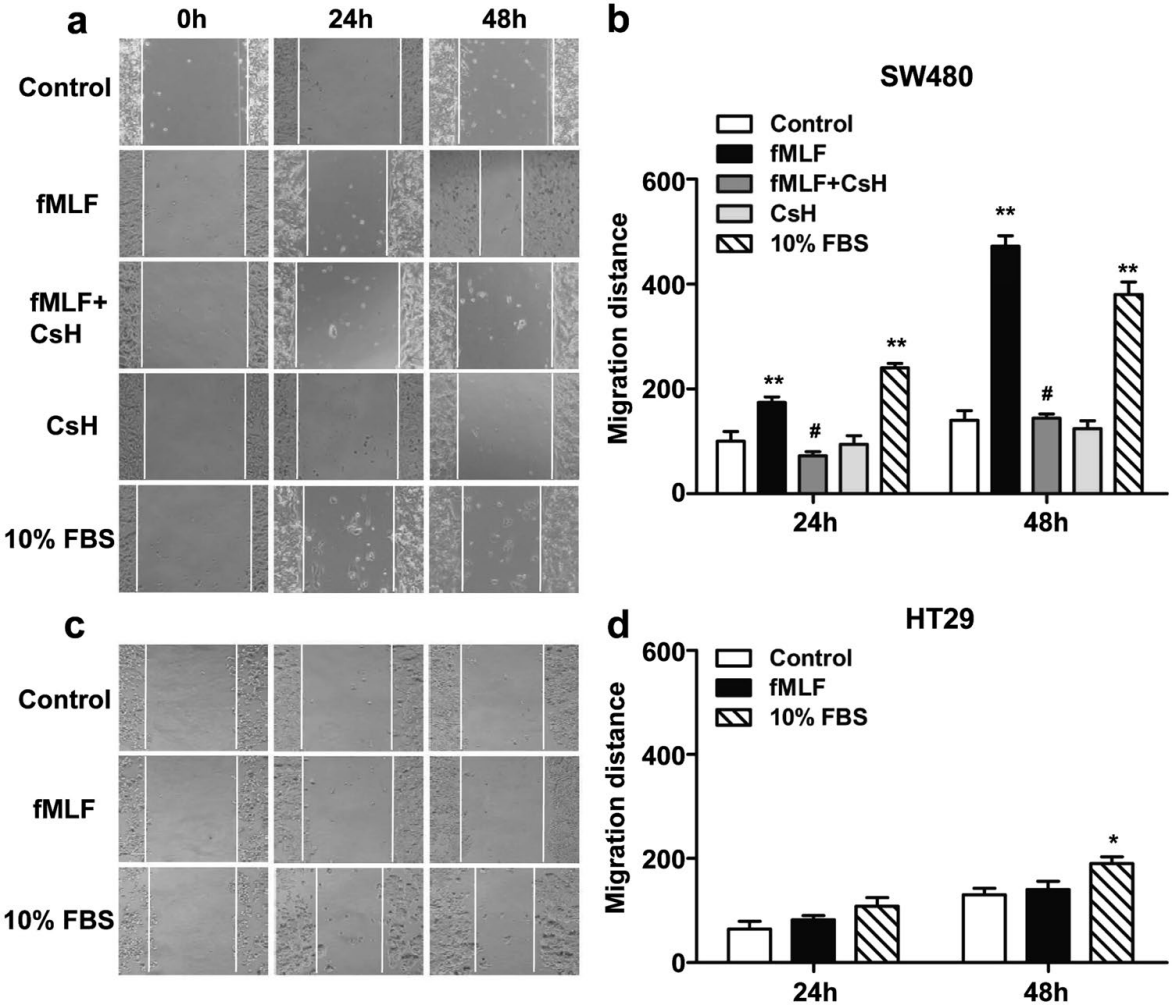
FPR1 activation accelerated the migration of SW480. The motility of SW480 (**a**,**b**) and HT29 (**c**,**d**) in wound-healing model was assayed in the presence of fMLF (1 μM) or 10% FBS with or without a 15 min pretreatment with cyclosporine H (1 μM). Then cells were photographed at 24 h and 48 h. *p < 0.05 compared with control, ^#^ *p* < 0.05 compared with the cells treated with fMLF. All the data shown are mean ± SEM from three separate experiments, each in triplicates.

To explore whether the activation of FPR1 plays a role in promoting the migration and invasion of CRC cells, Boyden chamber migration assay and wound healing assay were performed. For Boyden chamber migration assay, the cells were treated for 12 h with fMLF, an agonist of FPR1, with or without 15 min of pretreatment with the FPR1 antagonist cyclosporine H (1 μM) or Boc 1 (10 μM). A significant migration of SW480 cells was observed after treatment with fMLF, and pretreatment with cyclosporine H or Boc 1 reduced the migration of the cells (Fig. 4a and c and Supplementary Fig. S1), suggesting that the fMLF-induced migration was mediated through FPR1. In comparison, the fMLF-treated HT29 cells had no significant changes in migration (Fig. 4b and d). We further confirmed the chemotactic effect of fMLF on these cells using the wound healing assay. For wound healing assay, the cells were treated with fMLF for 24 h and 48 h, with or without a 15-min pretreatment with cyclosporine H (1 μM) or Boc 1 (10 μM). Images were taken and wound closure was quantified after 24 h and 48 h, respectively. Consistent with the result of the Boyden chamber migration assay, a significantly increased wound closure was found in SW480 cells treated with fMLF after 24 h and 48 h compared with cells treated with medium alone, and pretreatment with cyclosporine H or Boc 1 attenuated wound closure (Fig. 5a and b and Supplementary Fig. S2). As expected, the fMLF-treated HT29 cells showed no obvious wound recovery either at 24 h or 48 h (Fig. 5c and d), suggesting that activation of FPR1 by fMLF promotes SW480 cell migration.

### FPR1 was involved in survival of CRC mice

**Figure 6:**
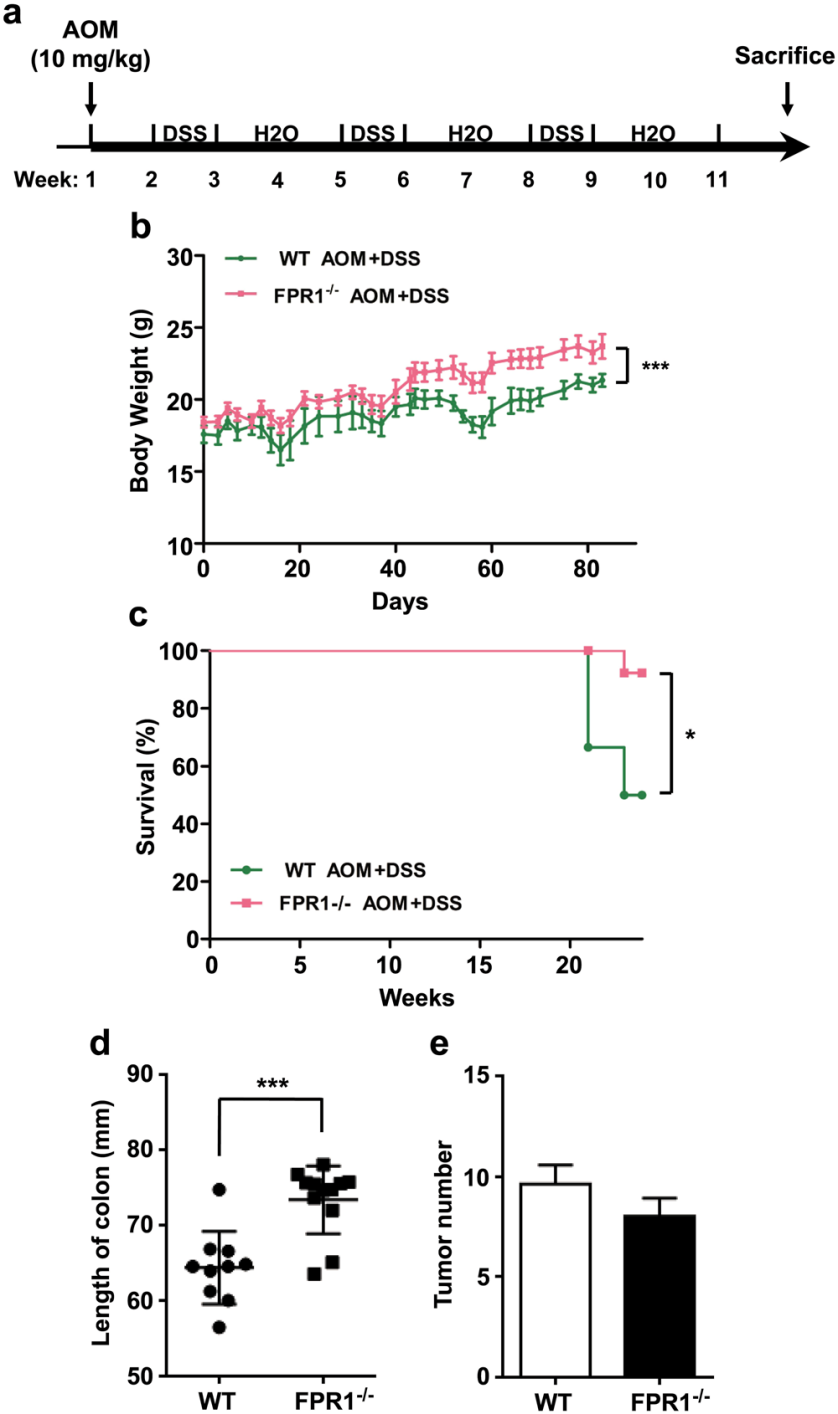
FPR1 involvement in mouse survival in a colorectal cancer model. (**a**) Schematic representation of the mouse model of CRC, as described in the *Materials and Methods* section. Body weights (**b**) and survival (**c**) of WT (green line) mice and *fpr1* ^−/−^ (red line) mice with CRC. Also shown are quantification of colon length (**d**) and tumor number (**e**) in WT mice and *fpr1* ^−/−^ mice with CRC. (6 mice for WT group; 13 mice for *fpr1* ^−/−^ group).

To assess the potential role of FPR1 in CRC *in vivo*, we established a CRC mouse model using WT and *fpr1* ^−/−^ mice. The combination of DSS (Dextran sulfate sodium) with AOM (Azoxymethane) is one of the well-known experimental models for colitis-associated CRC^25^. For AOM/DSS treatment experiment (Fig. 6a), as described in the *Materials and Methods* section, the body weight changes of the mice were monitored. Both WT mice and *fpr1* ^−/−^ mice had weight loss and the presence of diarrhea/hematochezia in the weeks following each DSS administration. However, the *fpr1* ^−/−^ mice showed marked recovery and gain of body weight afterwards compared with the WT mice (Fig. 6b). Furthermore, the survival rate of these mice was monitored. On week 21, two WT CRC mice died, and on week 23, one more WT CRC model mouse died; only one *fpr1* ^−/−^ CRC mouse died in the same period of time. By week 24, the survival rate of WT CRC mice was 50%, while that of *fpr1* ^−/−^ mice was 92% (Fig. 6c). This result indicates that depletion of *FPR1* markedly improved the survival rate of the CRC model mice. Furthermore, significantly reduced colon lengths were observed in WT mice when compared with *fpr1* ^−/−^ mice (Fig. 6d). However, there is no significant difference on the number of tumors between WT and *fpr1* ^−/−^ mice (Fig. 6e), suggesting that FPR1 may be involved in the progression, but not tumorigenesis of colorectal cancer.

## Discussion

FPRs were found in several types of human cancer tissues and were thought to have different functions in tumor growth and/or angiogenesis in different cancer histotypes^14–23^. In the present study, we found that the expression of FPR1 was associated with tumor serosal invasion. Our results also demonstrated that FPR1 activation promoted the migration and invasion of the CRC cell line SW480 in both the Boyden chamber migration assay and wound healing assay. Consistent with our findings, previous studies reported that high FPR1 expression was significantly associated with submucosal invasion and serosal invasion in gastric cancer^26^ and correlates with increased motility of human glioblastoma cells and the formation of highly invasive tumors^27^. Taken together, these data suggest that FPR1 play an important role in tumor invasion in colorectal cancer, gastric cancer, and glioblastoma.

In this work, we found that the expression of FPR1, but not FPR2 or FPR3, was associated with colorectal tumor serosal invasion. However, a recent work reported a correlation of FPR2 expression with the invasive phenotype of human colon cancer^28^. One of the differences between these studies is the use of different CRC tissues. In the published report, human colon cancer tissues were collected, while in our study both human colon cancer and rectal cancer tissues were used. Interestingly, we found FPR2 expression is significantly related with the location of the tumor (*p* = 0.002). There were 10 high FPR2 expressing cases (89%) from colon cancer patients, but just 1 (11%) from rectal cancer patient. Taken together, these results suggested a close correlation between FPR1 expression and colorectal cancers while FPR2 expression might be more sensitive in the colon cancer and affects the invasive phenotype of human colon cancer more specifically. It should be interesting to examine whether there is an exacerbated malignant phenotype of colon cancer co-expressing both FPR1 and FPR2 receptors.

The selected expression of FPR1 in glioblastoma cells and neuroblastoma cells has been investigated in previously studies^15, 21^. Zhou and colleagues demonstrated that FPR1 was selectively expressed in highly malignant human glioblastomas^15^. However, Snapkov *et al*. found that although the different neuroblastoma cell lines displayed differential expression of FPR1, there is no direct relationship between the expression of FPR1 and the various genetic aberrations and biological features of neuroblastoma^21^. In our study, FPR1 was moderately expressed in SW480 and HT29 at both mRNA and protein levels, using real-time PCR and western blot analysis respectively (Fig. 3). Since it is known that FPR1 expressed on cell surface can be activated by agonist binding, which then triggers a G protein-mediated signaling cascade, we further identified the cellular source and distribution of FPR1 in CRC. We examined its localization on the frozen slices of CRC patient tissues and on CRC cell lines, using immunofluorescence and flow cytometry analysis, respectively. We found that FPR1 was localized to the cytoplasm and plasma membrane of both primary colorectal epitheliums and tumor infiltrating myeloid cells, as well as in the two colorectal cell lines. Consistent with our study, Snapkov *et al*. reported that FPR1 was localized to the cytoplasm and plasma membrane of primary neuroblastoma tissues and cell lines^21^. However, another study reported that, in frozen sections of normal human colonic mucosa, FPR1 was identified in the crypt epithelium only in the lateral membrane, and no FPR1 protein was detected in surface enterocytes^29^. Several reasons may attribute to the difference between this report and our result. First, Babbin *et al*. used a monoclonal antibody (NFPR2) recognizing active FPR1^29^, while we used anti-FPR1 antibody recognizing all forms of FPR1. Another difference might be because we examined the expression of FPR1 in both primary CRC tissue and the adjacent normal tissue, while the above mentioned study only detected FPR1 in normal human colonic mucosa. Finally, the cited work considered that different expression of FPR1 might be attributed to differences in exposure to luminal formyl peptides, while in the present study both luminal formyl peptides and proinflammatory factors might affect the expression of FPR1 in a cancer environment.

Our data showed that the absence of FPR1 could increase the survival rate of *fpr1* ^−/−^ mice compared with WT mice in a mouse model of colitis-associated CRC, suggesting that FPR1 expression is a risk factor in the prognosis of CRC. This may result from the proinflammatory function of FPR1 especially in the infiltrating myeloid cells. At present, how FPR1 functions in colorectal epithelial cells remains unclear, but our results show that cancer cell migration correlates positively to cell surface expression of FPR1. These cells show clear polarity and the cell surface *vs*. intracellular expression of FPR1 is of potential interest, especially considering that regulated redistribution of this receptor may be associated with cell motility. The two CRC cell lines used in this study show different properties in chemotaxis and migration assays, which may be attributed to the fact that fewer receptors were detected in HT29 cells, although both cell lines express FPR1 on their cell surface. Consistent with our data, previous studies showed that high expression of FPR1 corresponded with poor survival in neuroblastoma and gastric cancer patients^21, 26^, and targeting FPR1 with an antagonist attenuated astrocytoma cell motility and prolonged the survival of tumour-bearing mice^16^. All these data demonstrate that FPR1 may play an important role in cancer development and invasion, and may represent a therapy option for the treatment of these neoplasms.

In summary, our studies have, for the first time, demonstrated that the expression of FPR1 is associated with tumor invasion of colorectal cancer. FPR1 are expressed both in the colorectal epitheliums and tumor-infiltrating myeloid cells. Furthermore, we confirm the function of FPR1 on tumor migration and invasion using chemotaxis and wound-healing assays in two colorectal cancer cell lines. Finally, we demonstrated that the expression of FPR1 is correlated with the survival of colitis-associated CRC mice. These findings provide a foundation for further investigation into the roles of FPR1 in cancer microenvironment, where myeloid cells are involved, and in the tumorigenic properties of colorectal epithelial cells such as its ability to metastasize to different tissues.

## Materials and Methods

### Tissue specimens and cell cultures

Twenty pairs of surgically resected CRC tissue specimens were obtained from Changzheng Hospital, Shanghai, China. Histological classification and stage of the CRC were determined according to the UICC TNM classification system^30^. Each patient signed the informed consent written in the Chinese language, which outlined the purpose and application of the colorectal tumor tissue that they donated. All samples were fixed with 10% formalin and cryoprotected in 30% sucrose for immunohistochemical staining or were kept frozen at −80 °C for other biochemistry analysis. Acquisition of human tissue specimens was approved by the Institution Review Board of Changzheng Hospital and was carried out in accordance with the Helsinki Declaration.

The two human CRC cell lines, SW480 and HT29, were obtained from ATCC (Manassas, VA). The cells were cultured in a humidified atmosphere of 95% air and 5% CO_2_ using Dulbecco’s modified Eagle’s medium (DMEM) supplemented with 10% fetal bovine serum (FBS) and antibiotics.

### Mice and Treatment

The FPR1 knock out (*fpr1* ^−/−^) mice in C57BL/6 background were kindly provided by Drs Philip Murphy and Ji-Liang Gao (NIAID, NIH, Bethesda, MD), as reported previously (Gao, J. L. *et al*., 1999). All mice were housed with a 12/12 h light/dark cycle with *ad libitum* access to food and water. The housing, breeding, and animal experiments were in accordance with the National Institutes of Health Guide for the Care and Use of Laboratory Animals, with procedures approved by the Biological Research Ethics Committee of Shanghai Jiao Tong University.

For AOM and DSS treatment experiment, female WT mice (n = 6) and *fpr1* ^−/−^ mice (n = 13) of 6 weeks in age were given a single intraperitoneal administration of AOM (Sigma-Aldrich, St. Louis, MO; 10 mg/kg body weight). Seven days later, these mice were fed with 1.5% DSS for 7 days (Sigma-Aldrich) in drinking water followed by two weeks of fresh water alternatively for 3 cycles as shown in Figure S2. The body weight of these mice was measured 2–3 times every week. The survival rate of these mice was monitored until 24^th^ week. Mouse survival curves were plotted as Kaplan-Meier plots. Statistical analysis was performed using the statistic software GraphPad Prism 5 (San Diego, CA) and *p* values less than 0.05 was considered statistically significant.

### Total RNA extraction and real-time PCR

The level of FPRs messenger RNA (mRNA) in tissues was determined using real-time quantitative PCR. Total RNA was extracted with Trizol reagent (Invitrogen, Carlsbad, CA) according to manufacturer’s instructions. One microgram of the RNA was used for reverse transcription using the Reverse Transcription System A3500 kit (Promega, Madison, WI). The complementary DNA (cDNA) was subsequently subjected to real-time PCR to quantify the transcripts of FPR1, FPR2 and FPR3 using SYBR^®^ Green Real-time PCR Master Mix (TOYOBO, Osaka, Japan). Primers were designed as follows: FPR1 (5′-CCTCCACTTTGCCATTC-3′; 5′-AGCAGAGCCATCACCC-3′), FPR2 (5′-GACACGCACAGTCACCA-3′; 5′-ACAGGAACCAGCCAAAA-3′), FPR3 (5′-TCTTTCAGTGCCATCCT-3′; 5′-ATCCAGAGTCCCGTCA-3′), GAPDH (5′-TCAAGAAGGTGGTGAAGCA-3′; 5′-AAAGGTGGAGGAGTGGGT-3′), and PPIA (5′-CCCCACCGTGTTCTTC-3′; 5′-GACCCGTATGCTTTAGGA-3′). PCR was performed according to the following conditions: 95 °C for 2 min; 40 cycles of denaturation at 95 °C for 15 s, annealing at 55 °C for 45 s, and extension at 72 °C for 30 s. A final extension step at 72 °C for 10 min was added before the hold step. The fold changes in mRNA level in each sample were normalized against the mRNA level of GAPDH/PPIA^24^ and calculated using the 2^exp(−ΔΔCt)^ method.

### Western Blot Analysis

Tissue specimens were homogenized in lysis buffer containing 50 mM Tris-HCl (pH7.4), 10 mM β-mercaptoethanol, 2 mM EDTA, 2 mM NaVanadate, 100 mM NaF, 8.5% sucrose, 5 μg/ml aprotinin, 5 μg/ml leupeptin, and 5 μg/ml pepstatin. Protein concentrations were determined using a BCA Protein Assay Kit (Beyotime Biotechnology, Nantong, China) and then heated in 5 × SDS-PAGE loading buffer (Beyotime Biotechnology) at 99 °C for 7 min. Tissue homogenates were separated on 12% SDS-PAGE and transferred onto nitrocellulose membranes (Whatman Protran, Dassel, Germany). Blots were blocked with 5% non-fat milk for 1 h at room temperature and incubated overnight at 4 °C with the primary antibody (anti-FPR1, BD Pharmingen, San Diego, CA; 1:1000) and anti-β-actin antibody (Cell Signaling Technology, Danvers, MA; 1:5000), followed by the respective IRDye^®^800CW secondary antibodies (LI-COR Biosciences, Lincoln, NE). The membranes were scanned using the 800-nm channel of an Odyssey^®^ CLX Infrared Imaging System (Li-COR Biosciences). The immunoreactive bands were quantified using the NIH Image J software (Bethesda, MD).

### Flow cytometry Analysis

The expression of FPR1 in CRC cell lines, SW480 and HT29, was detected using flow cytometry. The cells were harvested and resuspended in phosphate-buffered saline containing 1% bovine serum albumin on ice, then the cells were added the equal volume of 4% papaformaldehyde or fixation and Permeabilization solution (BD Pharmingen). Following incubation with purified mouse anti-FPR1 antibody (BD Pharmingen) for 1 h, the cells were incubated with FITC-conjugated anti-mouse secondary antibody in the dark for 30 min. Receptor density was analyzed as mean fluorescent intensity on a FACScan flow cytometer (BD Pharmingen). The results were expressed as the mean ± SEM based on at least three experiments.

### Immunofluorescence staining

Formalin-fixed, 30% sucrose solution-dehydrated tissue blocks of colorectal tumors with adjacent noncancerous colorectal mucosa were sliced into 40μm-thick frozen sections, as previously described^31^. Sections were incubated with a primary mouse monoclonal antibody against human FPR1 (BD Pharmingen) and a rabbit anti-myeloperoxidase (MPO) antibody (Cell Marque, Rocklin, CA) overnight at 4 °C, rinsed with TBS, and further treated with the secondary antibodies, Alex Fluor^®^488-conjugated anti-mouse antibody and Alex Fluor^®^568-conjugated anti-rabbit antibody (1:500, Invitrogen), respectively for 1 h in the dark at room temperature. Sections were stained for nuclei with 5 μg/mL 4,6-diamidino-2-phenylindole (DAPI, Beyotime Biotechnology) for 10 min, and then mounted on glass slides. Fluorescent images were taken on a laser-scanning confocal fluorescent microscope (Leica TCS SP8, Leica Microsystems, Wetzlar, Germany). The immunofluorescence intensity was quantified using the ImagePro Plus Software (Media Cybernetics, Silver Spring, MD). The results were expressed as means ± SEM based on a minimum of three individual fields.

### Chemotaxis assays

Chemotaxis assays were performed using a 48-well chemotaxis chamber (Neuro Probe, Gaithersburg, MD) according to the published procedures^32^. For SW480 and HT29 cells, after 12 h incubation with fMLF (1 μM), with or without a 15 min pretreatment with cyclosporine H (1 μM) or Boc1 (10 μM) at 37 °C, the filter was removed and fixed with methanol and stained with 0.1% crystal violet. Cells were counted in three random high-power fields in triplicate samples. The results were expressed as chemotaxis index which represents the fold increase in the number of migrated cells in response to chemoattractants over the response to control medium.

### Wound-healing assays

The wound-healing assays were performed as described previously^27^. The cells movement images were captured under light microscopy. Pictures were taken at 0 h as control. Then the cells were incubated with fMLF (1 μM) with or without 15 min pretreatment with cyclosporine H (1 μM) or Boc1 (10 μM) for 24 h or 48 h, and the pictures were taken at each of the time points. The results were quantified by calculating mean migrated distance of leading cells in the scratched area.

### Statistical analysis

All data are reported as the mean ± SEM. The expression of FPRs at transcript and protein levels were analyzed statistically for significant differences by one-way ANOVA followed by Newman-Keuls test. Correlations between the expressions of FPRs and various clinicopathological characteristics were analyzed using the Chi-square test. Statistical analysis was performed using the statistic software Prism (version 5, GraphPad, San Diego, CA) and *p* values less than 0.05 was considered statistically significant.

## Electronic supplementary material

Supplementary information
