# Supplementary material for: The Expression of Formyl Peptide Receptor 1 is Correlated with Tumor Invasion of Human Colorectal Cancer
Source: Sci Rep. 2017 Jul 19;7:5918. doi: 10.1038/s41598-017-06368-9 (PMC5517416; doi:10.1038/s41598-017-06368-9)
Supplement: Supplementary file 1 — Supplementary information [file 41598_2017_6368_MOESM1_ESM.pdf]

The Expression of Formyl Peptide Receptor 1 is Correlated with Tumor Invasion of Human Colorectal Cancer

Shu-Qin Li [1, 2, a], Ning Su [3, a], Ping Gong [1], Hai-Bo Zhang [1], Jin Liu [1], Ding Wang [1], Yan-Ping Sun [3], Yan Zhang [1], Feng Qian [1], Bo Zhao [1], Yang Yu [1, *], Richard D. Ye [4, *]

[1] *School of Pharmacy, Shanghai Jiao Tong University, 800 Dongchuan Road, Shanghai, 200240, China;*

[2] *School of Pharmacy, Ruijin Hospital Affiliated to Shanghai Jiao Tong University School of Medicine, 197 Ruijin Road (No.2), Shanghai, 200025, China;*

[3] *Department of General Surgery, Shanghai Chang Zheng Hospital, Second Military Medical University, 415 Fengyang Road, Shanghai, 200003, China*

[4] *Institute of Chinese Medical Sciences, University of Macau, Macau 999078, China*

Supplementary Materials

Figure S1

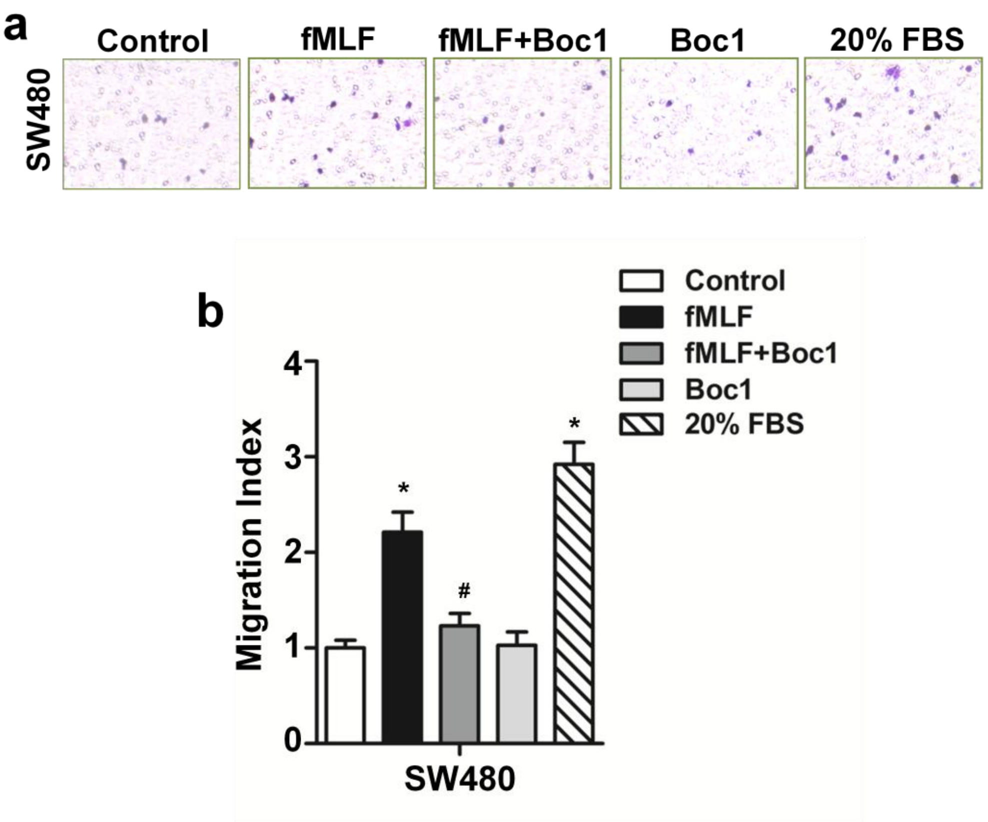

FPR1 activation promoted SW480 cell migration. SW480 (**a**) were treated with fMLF (1 μM) or 20% FBS (positive control) for 12 h with or without a 15 min pretreatment with Boc1 (10 μM). The cell migration was examined in a 48-well Boyden chamber. Representative images of migrated cells on membrane filters were shown in (**a**) and quantified data were shown in (**b**). * $p< 0.05$ compared with control, # $p< 0.05$ compared with the cells treated with fMLF. All the data shown are mean ± SEM from three separate experiments, each in triplicates.

Figure S2

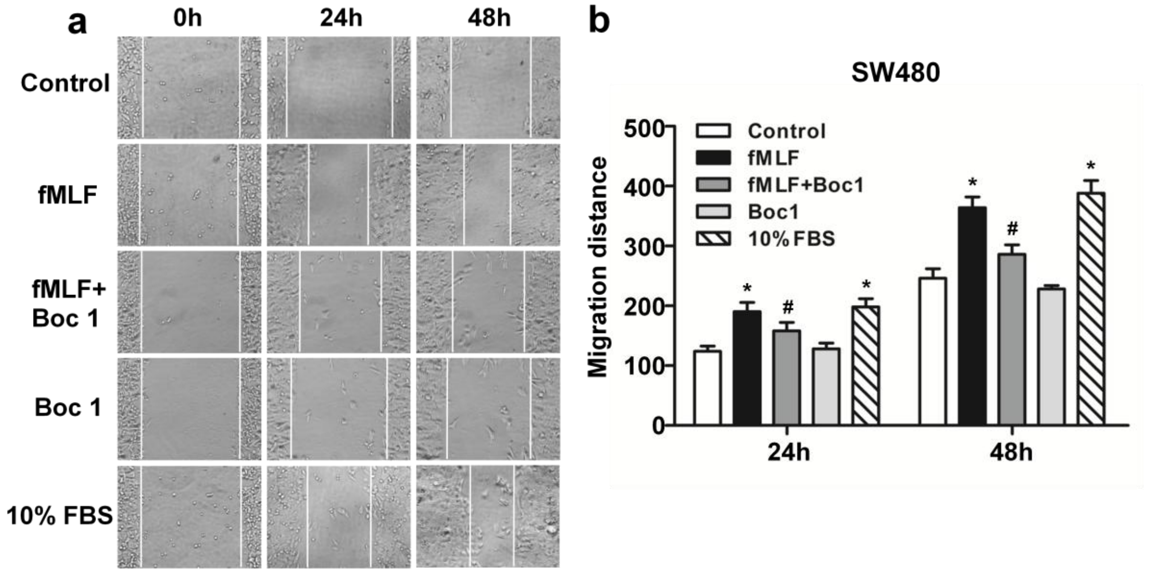

FPR1 activation accelerated the migration of SW480. The motility of SW480 in wound-healing model was assayed in the presence of fMLF (1 μM) or 10% FBS with or without a 15 min pretreatment with Boc 1 (10 μM). Then cells were photographed at 24 h and 48 h. * $p < 0.05$ compared with control, # $p < 0.05$ compared with the cells treated with fMLF. All the data shown are mean ± SEM from three separate experiments, each in triplicates.

Figure S3

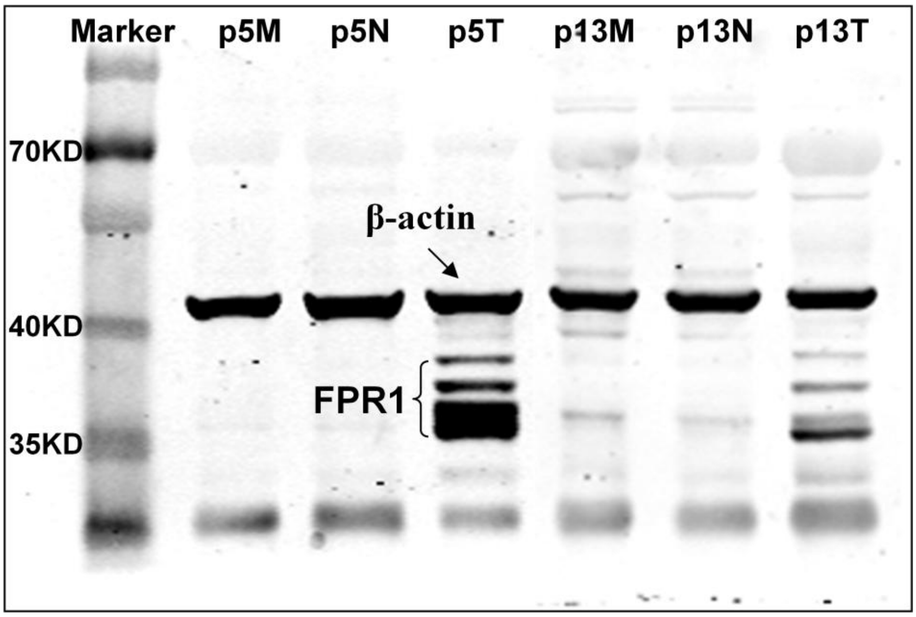

Full versions of western blots presented in figure 1b.

Figure S4

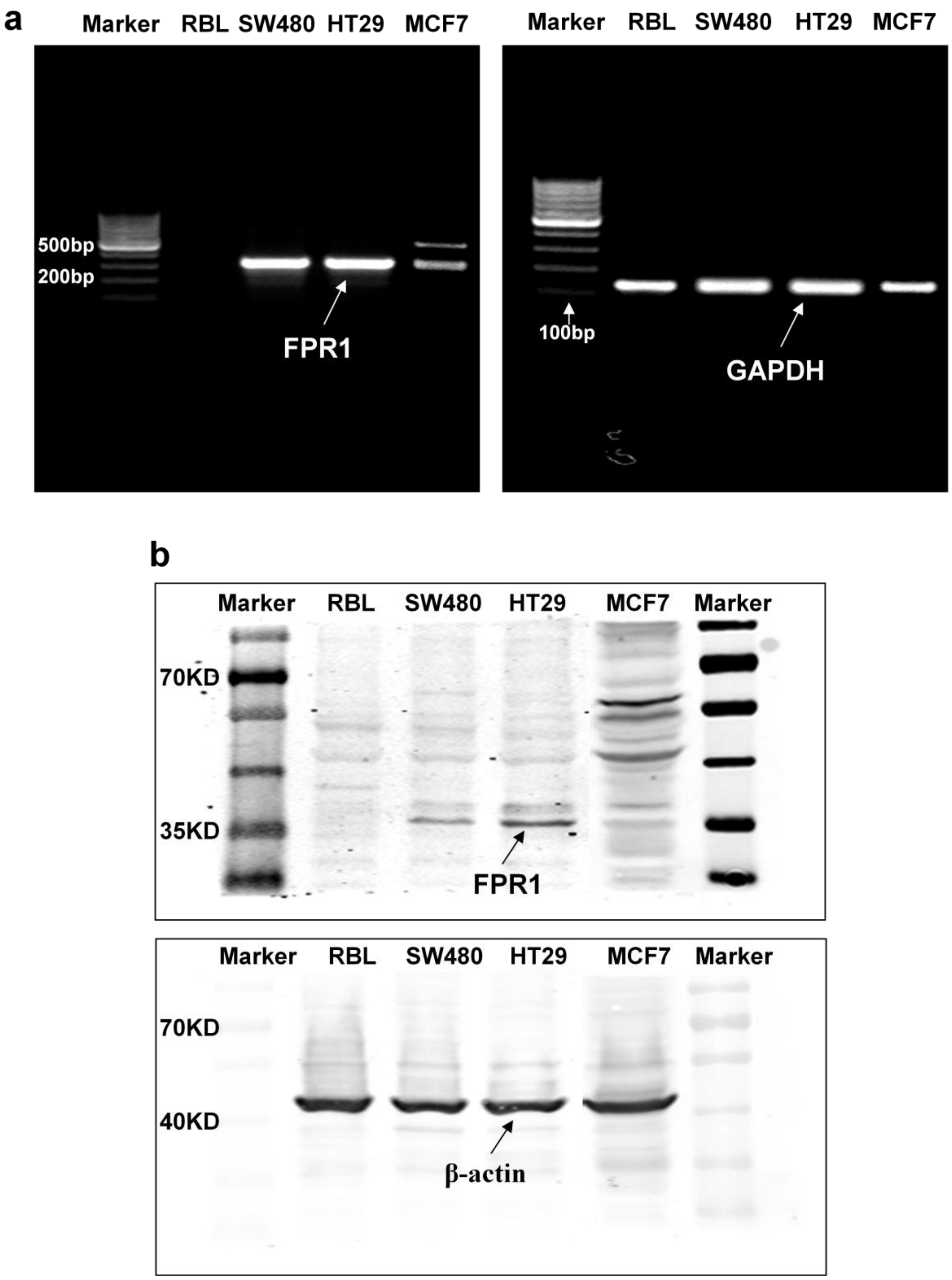

Full versions of gels and blots presented in figure 3a and 3b.

Table S1. Demographic and disease characteristics of colorectal cancer patients (n = 20)

Patient	Age (years)	Sex	Tumor Size (cm)	Site	Staging (TNM)	Clinical stage	Differenciation	Lymphatic invasion	Serosa infiltration	Distant Metastasis	FPR1	FPR2	FPR3
1	39	F	3.5	C	$T_3N_2M_0$	IIIB	Moderate	+	+	−	↑	↑↑	−
2	55	M	6.0	C	$T_4N_0M_0$	IIA	Moderate	−	+	−	↑	↑	↑
3	57	M	8.0	C	$T_4N_0M_1$	IV	Moderate	−	+	+	↑↑	↑	↑↑
4	60	F	5.0	C	$T_4N_2M_0$	IIIC	Moderate	+	+	−	↑↑	↑	↑↑
5	48	F	7.0	C	$T_4N_2M_0$	IIIC	Poor	+	+	−	−	↑	↑
6	74	F	6.5	R	$T_4N_2M_0$	IIIC	Poor	+	+	−	↑	−	−
7	45	M	7.0	C	$T_4N_0M_0$	IIA	Moderate	−	+	−	↑↑↑	↑↑	↑↑
8	49	M	5.5	R	$T_4N_2M_0$	IIIC	Moderate	+	+	−	↑	−	−
9	39	M	11.0	C	$T_3N_0M_0$	IIA	Moderate	−	+	−	↑↑	↑↑	↑
10	70	M	2.5	R	$T_2N_0M_0$	I	Moderate	−	+	−	−	−	↓
11	67	M	3.0	R	$T_{is}N_0M_0$	0	Good	−	−	−	↓	−	−
12	63	M	6.0	C	$T_4N_2M_0$	IIIC	Moderate	+	+	−	↑↑	↑	↑↑
13	68	F	3.5	R	$T_4N_1M_0$	IIIB	Moderate	+	+	−	↑↑	↑	↑
14	62	M	5.5	C	$T_2N_0M_0$	I	Moderate	−	−	−	↓	↑↑	−
15	55	F	3.0	R	$T_2N_0M_0$	I	Moderate	−	+	−	−	−	−
16	45	F	4.0	C	$T_4N_1M_0$	IIIB	Moderate	+	+	−	↓	↓	↓
17	59	F	3.5	R	$T_2N_0M_0$	I	Moderate	−	−	−	↓	−	↑
18	57	M	6.0	C	$T_3N_0M_0$	IIA	Poor	−	+	−	↑	−	↑
19	55	M	4.0	R	$T_4N_1M_0$	IIIB	Moderate	+	+	−	↑	−	↑
20	78	M	3.2	C	$T_4N_1M_0$	IIIB	Poor	+	+	−	↑	↑	−

M, Male; F, Female; C, Colon; R, Rectum
